# Predictive Value of the Alberta Stroke Program Early CT Score (ASPECTS) in the Outcome of the Acute Ischemic Stroke and Its Correlation with Stroke Subtypes, NIHSS, and Cognitive Impairment

**DOI:** 10.1155/2021/5935170

**Published:** 2021-01-29

**Authors:** Ahmed Esmael, Mohammed Elsherief, Khaled Eltoukhy

**Affiliations:** Neurology Department, Faculty of Medicine, Mansoura University, Egypt

## Abstract

**Objectives:**

This study is aimed at correlating ASPECTS with mortality and morbidity in patients with acute middle cerebral artery territory infarction and at determining the cutoff value of ASPECTS that may predict the outcome.

**Methods:**

150 patients diagnosed with acute middle cerebral artery territory infarction were involved in this study. Risk factors, initial NIHSS, and GCS were determined. An initial or follow-up noncontrast CT brain was done and assessed by ASPECTS. Outcomes were determined by mRS during the follow-up of cases after 3 months. Correlations of ASPECTS and outcome variables were done by Spearman correlation. Logistic regression analysis and ROC curve were done to detect the cutoff value of ASPECTS that predicts unfavorable outcomes.

**Results:**

The most common subtypes of ischemic strokes were lacunar stroke in 66 patients (44%), cardioembolic stroke in 39 patients (26%), and LAA stroke in 30 cases (20%). The cardioembolic stroke had a statistically significant lower ASPECT score than other types of ischemic strokes (*P* < 0.05). Spearman correlation showed that lower ASPECTS values (worse outcome) were more in older patients and associated with lower initial GCS. ASPECTS values were inversely correlated with initial NIHSS, inpatient stay, inpatient complications, mortality, and mRS. The ASPECTS cutoff value determined for the prediction of unfavorable outcomes was equal to ≤7. The binary logistic regression analysis detected that patients with ASPECTS ≤ 7 were significantly associated with about fourfold increased risk of poor outcomes (OR 3.95, 95% CI 2.09–11.38, and *P* < 0.01).

**Conclusions:**

ASPECTS is a valuable and appropriate technique for the evaluation of the prognosis in acute ischemic stroke. Patients with high ASPECTS values are more likely to attain favorable outcomes, and the cutoff value of ASPECTS is a strong predictor for unfavorable outcomes. This trial is registered with ClinicalTrials.gov NCT04235920.

## 1. Introduction

Stroke is a major etiology of long-term disability and is the second leading etiology of death worldwide [1]. The thirty-day mortality rate of ischemic strokes has been estimated at around 15% [2].

The ASPECTS was initially developed for the evaluation of the severity of early ischemic changes in cases with acute ischemic stroke (AIS) [3]. The ASPECTS evaluation has been increasingly involved in the choice of endovascular treatment [4, 5]. An ASPECTS value of 6 or more is now incorporated in the final version of the American Heart Association guidelines as an imaging qualification measure for endovascular treatment for cases presented in the time window (less than 6 hours) [6]. Previous studies demonstrated ASPECTS as an indicator of the outcome and suspected intracranial hemorrhage following intravenous thrombolytic therapy with a cutoff score equal to 7 or less as a potential indication of a higher risk of complications [7].

The TOAST classification is the most widely used system for establishing ischemic stroke etiology [8] and was further used for assessment of the prognosis of both ischemic stroke and TIAs [9, 10].

A high incidence of impaired cognition was detected in cases with ischemic strokes and was more likely a multifactorial process that includes the disturbance of the neuronal cellular networks [11]. The most common cognitive impairments among stroke survivors are memory, orientation, language and attention, executive dysfunction, and aphasia. Impairments of the cognitive functions are associated with higher morbidity and mortality, increased duration of hospital admission, more admissions to rehabilitation units, and unfavorable outcomes [12].

ASPECTS was widely used in clinical practice to determine the severity of ischemic injury on brain tomography for deciding the treatment of choice [7] and was reported to predict the neurological outcome; in particular, stroke patients with high ASPECTS have a more favorable prognosis and were treated with intravenous thrombolysis [13–15]. About 1% of patients who only presented AIS were treated with IV tPA [16]. Consequently, it will not express the true correlation of ASPECTS with different factors in patients analyzed within the ideal window time. The ASPECT score was originally designed for the identification of patients who were likely to show the most clinical benefit from intravenous thrombolysis [17]. But the aim of our study was unique compared to other studies as ASPECTS was used to investigate the correlation linking ASPECTS with the mortality and morbidity in cases of acute middle cerebral artery territory infarction, who presented within 2 days of the stroke onset and after the window time and not treated with intravenous thrombolysis or mechanical thrombectomy, and to determine the cutoff value of ASPECTS that may predict the outcome.

## 2. Patients and Methods

### 2.1. Selection of Participants

150 consecutive patients with AIS were admitted to the Convalescence Care Unit, Neurology Department (specialized stroke unit), between Oct 2017 and Mar 2019, retrospectively reviewed. Patients included 79 males and 71 females with an average age of 64.05 ± 11.55 and met the following inclusion criteria: (1) within 2 days from the onset, (2) the first attack of acute MCA territory infarction, and (3) aged more than 18 years.

Patients with recurrent stroke, presence of anterior or posterior cerebral artery infarction, and venous infarction were excluded, as well as patients presenting with major psychiatric illness, dementia, severe language impairment (aphasia), and previous CNS injury (e.g., brain tumor and traumatic brain injury).

### 2.2. Classifications and Patient Subgroups

According to mRS, patients were divided into two groups: the first one was a *good outcome* group with mRS 0-2, and the second group was a *poor outcome* group with mRS 3-6.

Patients were classified into two subgroups: the first group with *better ASPECTS* ranged from 10 to 8, and the second group with *worse ASPECTS* ranged from 0 to 7.

Also, patients were classified into two subgroups according to MoCA scores after 3 months of the onset of AIS. The first group was *cognitively impaired* with a MoCA score of 25 or less, and the second group was *cognitively preserved* with a MoCA score of higher than 25.

### 2.3. Neurological Examination, Clinical Scales, and Associated Comorbidities

Complete neurological and medical examinations were done for all patients. Through history taking, patients' age, sex, the onset of stroke, risk factors of stroke, initial NIHSS, and GCS scores were evaluated. NIHSS scores were divided into three categories, including mild (0–5), moderate (6–15), and severe (≥16) [18].

Cumulative Illness Rating Scale (CIRS) is a common scale used for assessing the associated comorbidities related to 14 body systems. Every system is evaluated on a five-point range (0-4). The maximum total score is 56. Higher scores indicate more severe comorbidities [19–21].

### 2.4. Image Acquisition and Analysis

All cases underwent CT brain (16-Multi-Slice GE, Optima 520, China). Initial noncontrast CT brain was carried out for all patients, and a follow-up CT brain was carried out after seven days. For all cases, the CT brain images were taken in an axial cut, 5 mm segments from the base to the vertex. The imaging parameters were 120 kVp, 320 mA, FOV 195 mm, and 1 s/rotation, and the speed of the table was 15 mm/rotation.

The ASPECTS format on noncontrast CT with 10 areas supplied by the middle cerebral artery territory at the ganglionic and supraganglionic levels was evaluated on all axial CT cuts. The cuts at the level or below the caudate head are considered the ganglionic level, while all cuts above the caudate head are considered the supraganglionic level [22].

**Figure 1:**
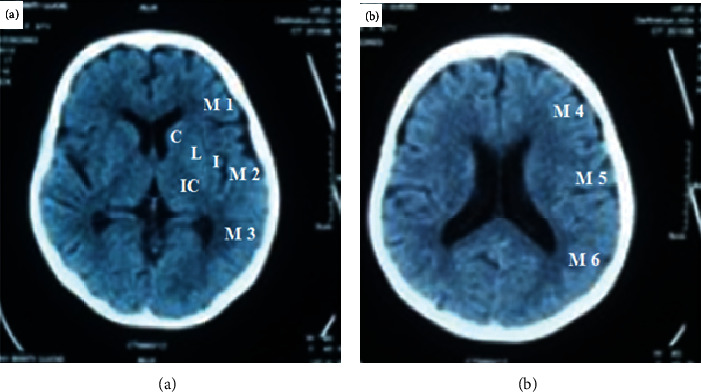
Alberta Stroke Program Early Computed Tomography Score template on noncontrast CT with 10 regions distributed over the MCA territory in (a) ganglionic and (b) supraganglionic levels. The template consists of 10 anatomically defined regions, 4 for subcortical structures (caudate (C), lentiform (L), internal capsule (IC), and insular ribbon (I)) and 6 for cortical structures in the MCA territory, labeled M1-M6.

The format comprises ten anatomically characteristic areas, four for subcortical areas (caudate, lentiform, internal capsule, and insular ribbon) and six for cortical areas in the middle cerebral artery territory, named M1, M2, M3, M4, M5, and M6 (Figure 1).

Early ischemic stroke signs on CT were known as areas of hypoattenuation and loss of gray-white matter differentiation that may be associated with focal swelling. In the ASPECTS zone that shows early ischemic changes affecting two successive cuts, the total value of ten is decreased by one. So, a value of zero means infarction affecting all ten areas.

Patients were subdivided into two groups according to the ASPECTS: the first group was better ASPECTS (8-10), and the second group was worse ASPECTS (7-0).

### 2.5. Stroke Subtypes

Our ischemic stroke patients were classified according to the TOAST classification into large vessel disease (large atherosclerosis), cardioembolic stroke, small vessel disease (lacunar stroke), and undetermined stroke [23].

#### 2.5.1. Large Artery Atherosclerosis

These patients have clinical and radiological imaging results of a significant (more than 50%) stenosis of a main cerebral artery or its branches, probably due to atherosclerosis. The sizes of infarcts are more than 1.5 cm in diameter on CT or MRI brain. Supportive proof by duplex imaging of stenosis of more than 50% of the intracranial or extracranial arteries is required. Sources of cardiogenic embolism should be excluded.

#### 2.5.2. Cardioembolism

This stroke subtype incorporates cases with artery occlusions, probably due to an embolus originating from the heart. Clinical and brain imaging results are comparable to those of large artery atherosclerosis. The possibility of large artery atherosclerotic origins of thrombosis or embolism should be excluded.

#### 2.5.3. Small Artery Occlusion (Lacunar Infarcts)

This category incorporates ischemic stroke cases frequently labeled as lacunar infarcts and has one of the classic clinical lacunar syndromes with no evidence of cerebral cortical dysfunction. The presence of diabetes mellitus or hypertension supports the diagnosis. The sizes of infarcts are less than 1.5 cm in diameter on CT or MRI brain.

#### 2.5.4. Acute Stroke of Other Determined Etiology

This stroke subtype incorporates cases with unusual etiology of strokes, such as nonatherosclerotic vasculopathy, hypercoagulation, or hematologic diseases showed by diagnostic studies like blood tests or arteriography. Cardioembolic stroke and large artery atherosclerosis should be excluded.

#### 2.5.5. Stroke of Undetermined Etiology

This stroke subtype incorporates cases with no likely etiology determined despite an extensive evaluation. It also incorporates cases with two or more suspected etiologies of stroke with the inability to make a final diagnosis.

### 2.6. Follow-Up of Cases for the Assessment of the Outcome and Cognition

Evaluation of the outcomes after 3 months was done by the modified Rankin Scale (mRS), which estimates the disability and can be utilized as a prognostic scale in stroke patients and divided into seven outcomes (0-6) [24]. Patients with better ASPECTS were compared with patients with worse ASPECTS according to mRS scores after 3 months of the onset of AIS.

Evaluation of the cognitive capacities of the study participants was carried out by the use of the Arabic form of the Montreal Cognitive Assessment (MoCA) [25]. This scale assesses distinctive domains of cognition of a total 30-point test [26]. Patients with better ASPECTS were compared with patients with worse ASPECTS according to the total MoCA scale after 3 months of the onset of AIS. Patients were considered to be cognitively impaired if the MoCA scores were ≤25 and cognitively preserved if the MoCA scores were ≥26.

### 2.7. Ethical Approval

Our study was approved by the Institutional Research Board (IRB) of the local ethical committee of Mansoura University, Faculty of Medicine.

### 2.8. Statistical Analysis

The data were analyzed by utilizing SPSS version 21. The data were demonstrated as mean ± SD and median. The comparative analysis between the two groups in case of continuous variables was done by using the one-way ANOVA or Kruskal-Wallis test, followed by the post hoc analysis test (if needed). Comparisons of the categorical data were made by using the chi-squared test. Spearman correlation was carried out to examine the relationship between ASPECTS and outcomes. Correlation linking ASPECTS and mRS was determined by using partial correlation coefficients (*r*). To detect the cutoff value for ASPECTS, a ROC curve was carried out. Lastly, logistic regression analysis was done to detect the predictive capability of the determined cutoff value as an independent variable. For all statistical analyses, *P* value of ≤0.05 was considered statistically significant. Logistic regression analysis was done to estimate the adjusted odds ratios and 95% confidence intervals for the prognostic value of the different risk factors of the outcome.

## 3. Results

### 3.1. Demographic and Clinical Data

150 patients of acute ischemic stroke with average age 64.0 ± 11.5 included 79 (52.7%) males and 71 (47.3%) females. The most common risk factors were hypertension (68%), smoking (40%), diabetes mellitus (26%), atrial fibrillation (18.6%), hyperlipidemia (14.6%), and coronary heart disease (10%). The average initial NIHSS was 12.9 ± 7, and the mean ASPECTS was 6.82 ± 2.32. Higher age, hypertension, higher NIHSS, and lower ASPECTS were significantly associated with poor outcomes (higher mRS). Sex, AF, DM, hyperlipidemia, IHD, and smoking did not vary significantly regarding the good and poor outcomes (Table 1).

### 3.2. ASPECTS according to Stroke Subtypes

Table 2 shows that the foremost common sorts of ischemic strokes in our study were lacunar stroke in 66 patients (44%), cardioembolic stroke in 39 patients (26%), and LAA stroke in 30 cases (20%).

**Figure 2:**
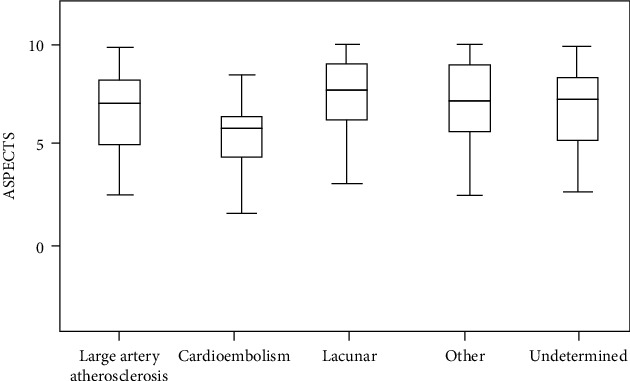
ASPECTS according to stroke subtypes (*P* < 0.05).

The cardioembolic stroke had a low ASPECTS value, which was less than other ischemic stroke subtypes (LAA, lacunar, and undetermined etiology) and which was statistically significant (*P* < 0.05) (Figure 2).

### 3.3. Relationship of ASPECTS with Stroke Severity, Primary Outcomes, and Morbidity

**Figure 3:**
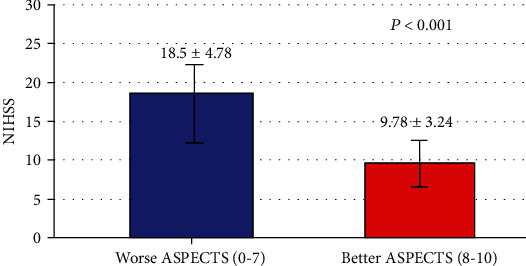
Relationship of ASPECTS with NIHSS.

Table 3 shows that low scores of ASPECTS (worse ASPECTS) compared with high scores of ASPECTS (better ASPECTS) were significantly associated with severe NIHSS (*P* < 0.0001), more inpatient stay (*P* < 0.005), more inpatient complications, and lower GCS (*P* < 0.05). In addition, low scores of ASPECTS (worse ASPECTS) compared with high scores of ASPECTS (better ASPECTS) were significantly associated with higher total NIHSS (18.5 ± 4.78 versus 9.78 ± 3.24, *P* < 0.0001) (Figure 3) and higher Cumulative Illness Rating Scale (14 ± 4.5 versus 11 ± 4.6, *P* < 0.05).

### 3.4. Association of ASPECTS with Mortality and Secondary Outcomes

**Figure 4:**
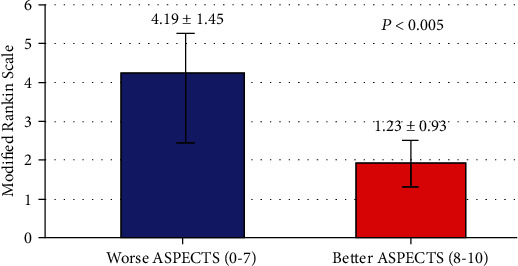
Relationship of ASPECTS with the modified Rankin Scale.

Table 4 demonstrates that the mortality after 3 months was 20 cases (13.3%). Low scores of ASPECTS (worse ASPECTS) compared with high scores of ASPECTS (better ASPECTS) were significantly associated with a higher mortality rate (17 (20.2%) versus 3 (4.5%), *P* = 0.005). In addition, low scores of ASPECTS (worse ASPECTS) were significantly associated with poorer outcomes and disability as determined by mRS compared with high scores of ASPECTS (better ASPECTS) (4.19 ± 1.45 versus 1.23 ± 0.93, *P* < 0.005) (Figure 4).

### 3.5. Cognitive Impairment in Patients according to ASPECTS

Worse ASPECTS values were more common with older patients (67.94 ± 9.23, *P* = 0.001) compared with better ASPECTS, with no sex difference between both groups. Worse ASPECTS values compared with better ASPECTS values were significantly associated with lower total MoCA score (23.32 ± 4.75 versus 26.54 ± 3.43, *P* < 0.005), lower executive functions (*P* < 0.005), lower attention (*P* = 0.03), lower language (*P* = 0.03), and lower memory (*P* < 0.005). There was no significant difference regarding the level of education (*P* > 0.05) between both groups (Table 5).

### 3.6. Correlations of ASPECTS with Morbidity and Mortality

Spearman correlation showed that lower ASPECTS values (worse outcome) were more in older patients (*r* = −0.70 and *P* = 0.001) and associated with lower initial GCS (*r* = 0.56 and *P* < 0.05). ASPECTS values were inversely correlated with initial NIHSS (*r* = −0.75 and *P* < 0.001), inpatient stay (*r* = −0.72 and *P* = 0.005), inpatient complications (*r* = −0.60 and *P* = 0.01), mortality (*r* = −0.73 and *P* = 0.005), and mRS (*r* = −0.74 and *P* < 0.005) (Table 6).

### 3.7. Prediction of the Development of Unfavorable Outcomes according to ASPECTS

**Figure 5:**
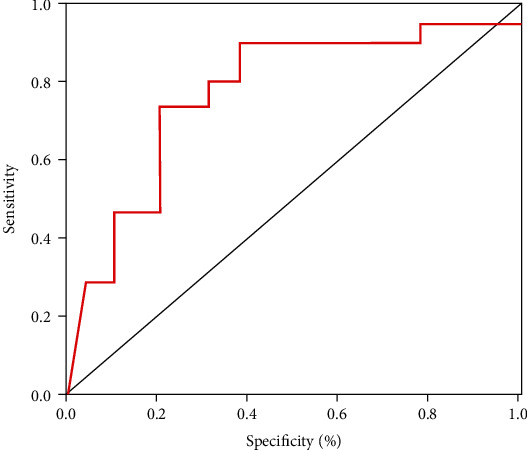
ROC curve for estimation of cutoff scores of ASPECTS for the outcome of acute ischemic stroke.

Table 7 shows that the ASPECTS cutoff value determined for the prediction of unfavorable outcomes attains a prominent sensitivity and specificity of more than seventy percent. The calculated cutoff score for ASPECTS was ≤7. The total performance of the cutoff scores was detected by the ROC curve (Figure 5). A binary logistic regression analysis detected that ASPECTS ≤ 7 was significantly associated with about fourfold increased risk of unfavorable outcomes (OR 3.95, 95% CI 2.09–11.27, and *P* < 0.01).

### 3.8. Prognostic Model Using a Regression Analysis with mRS

A prognostic model using a regression analysis with mRS showed that the independent factors accompanied by poor outcomes were older age (OR 2. 11, *P* = 0.001), higher initial NIHSS (OR 2. 34, *P* < 0.001), and higher Cumulative Illness Rating Scale (OR 2. 31, *P* < 0.001), followed by HTN and lower ASPECTS (OR 1.96, *P* = 0.005 and OR 1.89, *P* = 0.005, respectively), and lastly lower initial GCS, lower total MoCA score, and cardioembolic stroke subtype (OR 1. 25, *P* < 0.05; OR 1. 56, *P* = 0.01; and OR 1. 23, *P* < 0.05, respectively) (Table 8).

## 4. Discussion

AIS needs rapid clinical and radiological assessment. The ability to distinguish an acute infarct by CT is helpful in confirming the diagnosis and analysis of acute stroke [27]. CT has the advantage of being a simple technique and can spare time for early treatment and fast intervention if needed. The baseline ASPECTS is a reliable predictor of the prognosis in patients with AIS [28]. ASPECTS has been included in the decision-making and assessment of neurovascular interventions in patients with AIS [3]. In the present study, we try to correlate ASPECTS with stroke subtypes, outcomes, and cognitive impairment in AIS.

In our study, the most common risk factors for AIS were hypertension (68%), smoking (40%), diabetes mellitus (26%), atrial fibrillation (18.6%), dyslipidemia (14.6%), coronary heart disease (10%), and recurrent stroke (6.7%). These classical risk factors were similar to the study of Boehme et al. and Habibi-Koolaee et al. [29, 30].

### 4.1. ASPECTS and Stroke Subtypes

The foremost common sorts of ischemic strokes in our study were lacunar stroke in 66 patients (44%), cardioembolic stroke in 39 patients (26%), and LAA stroke in 30 cases (20%).

The distribution of stroke subtypes is similar to that detected by Kim and his colleagues, who stated that lacunar stroke had a higher incidence in Asia than other subtypes of stroke [31]. Acute large vessel occlusions were found in 28.7% of cases with hyperacute cerebral ischemic infarction in the study of Hansen and his colleagues [32], and approximately 25% of ischemic strokes were of cardioembolic origin [33, 34].

Our study detected that cardioembolic strokes had lower ASPECTS values (6.34 ± 1.37) than LAA strokes (7.36 ± 1.09), lacunar strokes (8.46 ± 1.04), strokes due to other causes (8.12 ± 1.12), and strokes due to undetermined cause (8.34 ± 1.32). This is similar to a study carried out by Horie and his colleague, who demonstrated that cardioembolic strokes had larger infarct lesions on MRI-DWI in comparison with LAA and lacunar strokes [35]. Cardiac emboli tend to have a larger volume than emboli arising from the carotid arteries; consequently, the embolic can obstruct the arteries in a proximal site of the affected artery, causing a larger infarction size [34–36].

The estimate of ischemic infarctions due to lacunar strokes is less than 1.5 mm in size and tends to be within the penetrating arteries of the middle cerebral artery [36, 37]. Ischemic infarctions in LAA are caused by artery-to-artery emboli, and the estimates of these emboli are lesser than those of the cardiac emboli so that they cause an obstruction in the distal arterial sites and the severity of the resulting acute ischemic injury is lesser [35].

### 4.2. The Mortality Rate of AIS after 3 Months

The mortality rate after 90 days in our study was 13.3%. These results are similar to a large retrospective study that involved 12,262 cases of acute ischemic infarction at multiple hospitals in Ontario, Canada; the mortality rates were about 12.2% at 30 days [38]. On the other hand, stroke unit treatment is regarded as the gold standard of acute stroke care and has been consistently associated with lower mortality rates, irrespective of patients' age or clinical characteristics [39–41].

### 4.3. Correlation of ASPECTS with NIHSS

In our study, ASPECTS demonstrated an inverse correlation with initial NIHSS in ischemic stroke, which was similar to the findings of Kent et al., who detected a powerful, inverse relationship linking initial NIHSS and ASPECTS; and every increase of ten points on initial NIHSS was associated to a decrease of about three points on ASPECTS [42]. This is in accordance with Amalia et al., who concluded that the higher ASPECTS values in acute ischemic infarction had lower NIHSS scores and vice versa [43]. Also, the findings of Hill found that cases with ASPECTS equal to 6-10 points have a higher future of independent living and better outcomes [44].

### 4.4. Correlation of ASPECTS with Outcomes

Patients with lower ASPECTS ≤ 7 (worse ASPECTS group) were accompanied by low GCS, high initial NIHSS, increased inpatient admission, and higher incidence of inpatient complications. The use of CT scans by using ASPECTS can help in predicting stroke outcomes and management [45, 46]. However, González and his colleagues showed no significant predictive value of ASPECTS on initial noncontrast CT carried out after 24 hours from the initial onset of symptoms in 649 cases of ischemic infarction [47].

Patients who presented with minor ischemic stroke usually recover rapidly with minimal or without inpatient complications. So, cases with better ASPECTS (8-10) usually recover sooner with fewer inpatient complications with shorter durations of hospitalization. Also, ASPECTS of 8-10 points was accompanied by lower NIHSS and higher GCS at admission, indicating a minor stroke, which demonstrated good outcomes (≤3 mRS). In addition, ASPECTS is a good predictor of mortality. These results agree with previous studies [48–50].

Our results demonstrated that older age, hypertension, more severe NIHSS, and lower ASPECTS were significantly associated with unfavorable outcomes, while a large Swedish study of 15,959 stroke patients demonstrated that higher mRS, male, higher age, DM, smoking, HTN, AF, and depressed mood were significant predictors of unfavorable outcomes [51]. Generally, larger strokes associated with more severe initial NIHSS and lower ASPECTS have unfavorable outcomes compared with smaller strokes associated with less severe initial NIHSS and higher ASPECTS [52].

### 4.5. Prediction of the Development of Unfavorable Outcomes according to ASPECTS

Our study showed that the ASPECTS cutoff value determined for the prediction of unfavorable outcomes achieved sensitivity and specificity greater than 70%, which was equal to ≤7. Consequently, patients with a score of more than seven in CT ASPECTS were associated with a favorable prognosis [53]. The binary logistic regression analysis detected that patients with ASPECTS ≤ 7 were significantly associated with about fourfold increased risk of poor outcomes (OR 3.95, 95% CI 2.09–11.38, and *P* < 0.01).

mRS and the functional outcome following the ischemic stroke are dependent on different factors, like age, sex, comorbid diseases, the severity of the neurological insult, the subtypes of stroke, and the management and treatment prior to and during admission to the hospital [54–56]. So, the combinations of variable factors for the prediction of poor outcomes have been determined [57–62]. Age and the severity of the neurological deficits are considered major factors, which is consistent among different studies, while the linkage of other factors with the functional outcomes was variable across different studies [57–62]. In our study, a prognostic model using a regression analysis with mRS showed that the independent factors accompanied by poor outcomes were older age, higher initial NIHSS, and higher Cumulative Illness Rating Scale, followed by HTN and lower ASPECTS, and lastly lower GCS, lower total MoCA score, and cardioembolic stroke subtypes.

## 5. Conclusion

ASPECTS is a valuable and appropriate technique for the assessment of the severity and prognosis of acute ischemic lesions. The cardioembolic stroke had a low ASPECTS value, which was less than other ischemic stroke subtypes. Patients with high ASPECTS values are more likely to attain favorable outcomes. According to the present study, a clear cutoff value of ASPECTS ≤ 7 is a strong predictor for unfavorable outcomes.

## Figures and Tables

**Table 1:** Characteristics of patients with acute ischemic stroke and classification of patient outcomes according to mRS after 3 months.

Variable	Total patients	Good outcome	Poor outcome	*P* value
mRS	0-6	0-2	3-6	
Number	150 (100%)	95 (63.3%)	55 (33.7%)	*P* = 0.05
Age (years)	64.0 ± 11.5	59.11 ± 7.45	68.7 ± 6.98	*P* = 0.001^∗^
Male	79 (52.7%)	49 (51.5%)	30 (54.5%)	*P* = 0.725
Hypertension	102 (68%)	54 (56.8%)	48 (87.3%)	*P* = 0.005^∗^
DM	39 (26%)	23 (24.2%)	16 (29.1%)	*P* = 0.511
Smoking	60 (40%)	37 (38.9%)	23 (41.8%)	*P* = 0.729
Hyperlipidemia	22 (14.6%)	13 (13.7%)	9 (16.4%)	*P* = 0.654
AF	28 (18.6%)	17 (17.9%)	11 (20%)	*P* = 0.749
IHD	15 (10%)	10 (10.5%)	5 (9.1%)	*P* = 0.778
Initial NIHSS	12.9 ± 7	10.97 ± 4.64	18.92 ± 6.32	*P* = 0.001^∗^
ASPECTS	6.82 ± 2.32	8.23 ± 1.87	4.96 ± 2.56	*P* = 0.001^∗^

*P* value for the comparison between the good outcome and poor outcome groups. ^∗^Significant.

**Table 2:** ASPECTS according to stroke subtypes.

Stroke subtypes	Number of patients (150)	ASPECTS	*P* value
Large artery atherosclerosis	30 (20%)	7.36 ± 1.09	*P* < 0.05
Cardioembolism	39 (26%)	6.34 ± 1.37	
Lacunar	66 (44%)	8.46 ± 1.04	
Other	6 (4%)	8.12 ± 1.12	
Undetermined	9 (6%)	8.34 ± 1.32	

**Table 3:** Relationship of ASPECTS with NIHSS, early outcomes, and morbidity.

Outcome	Better ASPECTS	Worse ASPECTS	*P* value
	8-10 (*N* = 66)	0-7 (*N* = 84)	
Total NIHSS	9.78 ± 3.24	18.5 ± 4.78	*P* < 0.001
Mild NIHSS (0–5)	27	2	*P* < 0.0001
Moderate NIHSS (6–15)	23	17	
Severe NIHSS (≥16)	16	65	
GCS at admission	13.77 ± 1.23	9.05 ± 3.42	*P* < 0.05
Inpatient stay (days)	3.65 ± 4.87	13.39 ± 6.94	*P* < 0.005
Inpatient complications	10 (15.1%)	27 (32.1%)	*P* = 0.01
Cumulative Illness Rating Scale	11 ± 4.6	14 ± 4.5	*P* < 0.05

**Table 4:** Relationship of ASPECTS with mortality and delayed outcomes (mRS).

Outcome	Better ASPECTS	Worse ASPECTS	*P* value
	8-10 (*N* = 66)	0-7 (*N* = 84)	
Mortality			
Total (*N*) 20 (13.3%)	3 (4.5%)	17 (20.2%)	*P* = 0.005
Modified Rankin Scale after 3 months	1.23 ± 0.93	4.19 ± 1.45	*P* < 0.005

**Table 5:** Patients with cognitive impairment compared with patients with preserved cognition.

	Better ASPECTS	Worse ASPECTS	*P* value
Number	66 (44%)	84 (56%)	
Age (years)	61.06 ± 7.36	67.94 ± 9.23	*P* = 0.001^∗^
Sex (male/female)	32/34	47/37	*P* = 0.36
Education			
Primary school	14 (21.2%)	21 (25%)	*P* = 0.69
Secondary school	16 (24.2%)	19 (22.6%)	
Tertiary school	17 (25.8%)	26 (31%)	
University education	19 (28.8%)	18 (21.4%)	
MoCA test scores
Visual-spatial ability	3.38 ± 0.95	3.09 ± 1.14	*P* = 0.31
Naming	2.49 ± 0.61	2.31 ± 0.88	*P* = 0.24
Executive functions	3.64 ± 0.89	2.31 ± 1.18	*P* < 0.005^∗^
Attention	5.75 ± 1.23	4.42 ± 1.32	*P* = 0.03^∗^
Language	4.39 ± 1.21	3.39 ± 1.11	*P* = 0.02^∗^
Memory	3.69 ± 1.32	2.91 ± 1.36	*P* < 0.005^∗^
Orientation	6.14 ± 0.49	5.75 ± 0.24	*P* = 0.13
Total MoCA score	26.54 ± 3.43	23.32 ± 4.75	*P* < 0.005^∗^

**Table 6:** Correlations of ASPECTS with morbidity and mortality in acute ischemic stroke.

	*r*	*P*
Age	-0.70	*P* = 0.001
Initial NIHSS	-0.75	*P* < 0.001
Initial GCS	0.56	*P* < 0.05
Inpatient stay	-0.72	*P* = 0.005
Inpatient complications	-0.60	*P* = 0.01
Mortality	-0.73	*P* = 0.005
Modified Rankin Scale	-0.74	*P* < 0.005

**Table 7:** Cutoff score of ASPECTS and prediction of the outcome of acute ischemic stroke.

Variable	Value
Cutoff score	≤7
Sensitivity	0.73
Specificity	0.81
PPV	0.87
NPV	0.73
OR	3.95
95% CI	2.09–11.28
*P* value	*P* < 0.01

**Table 8:** Prognostic model using a regression analysis with mRS.

Variables	OR	95% CI	*P*
Age	2.11	1.54–2.85	*P* = 0.001
HTN	1.96	1.29–2.59	*P* = 0.005
Initial NIHSS	2.34	1.74–3.04	*P* < 0.001
Initial GCS	1.25	1.02–1.71	*P* < 0.05
ASPECTS	1.89	1.26–2.47	*P* = 0.005
Total MoCA	1.56	1.14–2.14	*P* = 0.01
Cumulative Illness Rating Scale	2.31	1.69–3.02	*P* < 0.001
Cardioembolic stroke	1.23	0.95–1.73	*P* < 0.05

## Data Availability

All the data used to support the findings of this study are included within the article.
